# Outcomes of acute myeloid leukemia patients undergoing allogeneic hematopoietic stem cell transplantation: validation, comparison and improvement of 2022 ELN genetic risk system

**DOI:** 10.1186/s40164-024-00487-6

**Published:** 2024-02-15

**Authors:** Haixiao Zhang, Xinhui Zheng, Wenwen Guo, Yonghui Xia, Rongli Zhang, Weihua Zhai, Xin Chen, Qiaoling Ma, Donglin Yang, Jialin Wei, Aiming Pang, Yi He, Sizhou Feng, Jianxiang Wang, Mingzhe Han, Erlie Jiang

**Affiliations:** 1 State Key Laboratory of Experimental Hematology, National Clinical Research Center for Blood Diseases, Haihe Laboratory of Cell Ecosystem, Institute of Hematology & Blood Diseases Hospital, Chinese Academy of Medical Sciences & Peking Union Medical College, Tianjin, China; 2 Hematopoietic Stem Cell Transplantation Center, Institute of Hematology & Blood Diseases Hospital, Chinese Academy of Medical Sciences & Peking Union Medical College, Tianjin, China

## Abstract

The 2022 European LeukemiaNet (ELN) updated the previous risk classification published in 2017 but the prognostic significance for allogeneic hematopoietic stem cell transplantation (allo-HSCT) remains unclear. We enrolled 600 acute myeloid leukemia (AML) patients who underwent allo-HSCT to validate ELN-2022 genetic risk system and compared it with ELN-2017. There were 214 (35.67%), 162 (27.0%), and 224 (37.33%) patients in ELN-2022 favorable-, intermediate-, and adverse-risk group respectively and 86 patients (14.33%) experienced a shift in risk stratification compared to ELN-2017. Median and maximum follow-up time were 2.89 (95% CI 2.67 to 3.03) years and 8.78 years. The median overall survival (OS) was 73.8% (95% CI 67.5% to 80.3%), 63.9% (95% CI 56.7% to 72.0%) and 57.6% (95% CI 50.4% to 65.9%) in ELN-2022 favorable-, intermediate-, and adverse-risk group (P < 0.001). OS shortened significantly as the ELN-2022 risk stratification increased but didn’t significantly in ELN-2017 intermediate-risk compared to favorable-risk. Both ELN-2022 and ELN-2017 adverse-risk were associated with increased cumulative incidence of relapse (CIR). Time-dependent receiver operating characteristic (ROC) analysis showed that both ELN-2017 and ELN-2022 risk systems had limited prognostic ability for OS. We modified ELN-2022 risk system with pre-transplant minimal residual disease (MRD) and the modified risk system performed a significantly superior efficacy to ELN-2022 system.

**Supplementary Information:**

The online version contains supplementary material available at 10.1186/s40164-024-00487-6.

## To the editor

Advancements in understanding acute myeloid leukemia (AML) genetics have led to new diagnostic entities and improved prognostic system [1–4]. The European LeukemiaNet (ELN) group updated prognostic stratification in 2022, which has been validated in several chemotherapeutic AML cohorts [5–8]. However, the applicability of ELN-2022 risk system in AML patients undergoing allogeneic hematopoietic stem cell transplantation (allo-HSCT) remains uncertain. Our study aims to shed light on this.

**Fig. 1:**
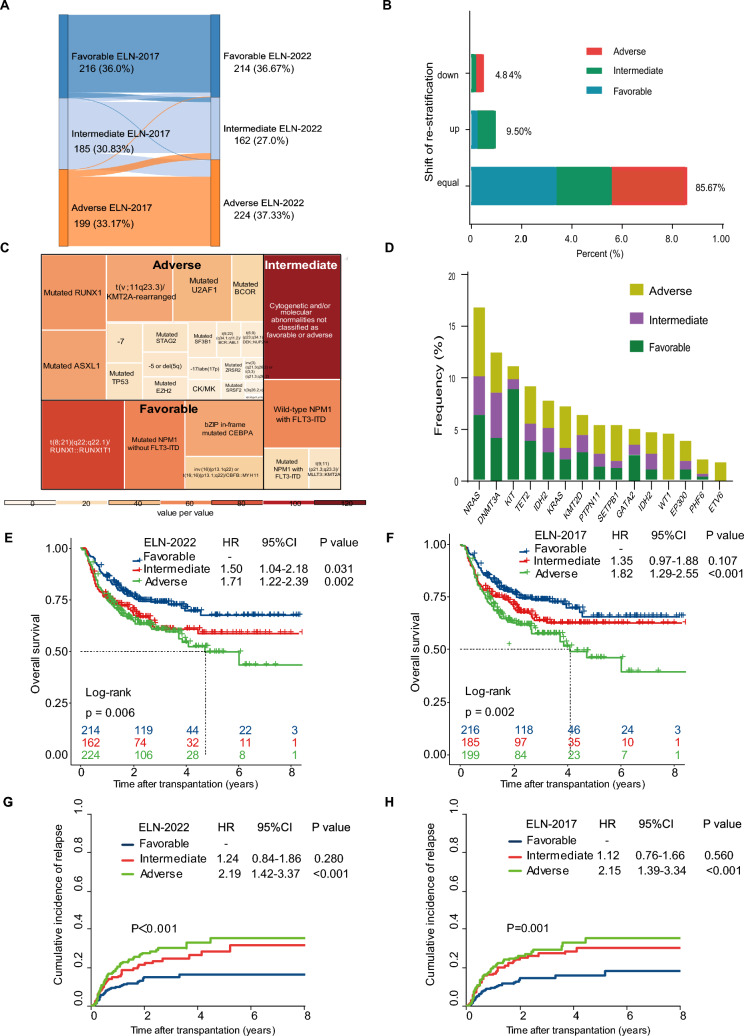
Patients and genetic characteristics and impact of ELN-2022 and ELN-2017 risk stratification on clinical outcomes. **A** Relationship of risk groups between ELN-2022 and ELN-2017 risk groups; **B** Distribution of re-stratification in ELN-2017 risk groups. **C** Landscape of genetic abnormalities defined by ELN-2022 genetic risk categories. The color scale is representative of a number of patients. **D** Additional mutations stratified by ELN-2022 genetic risk categories. Genes mutated in more than ten patients are shown. **E** Overall survival stratified by ELN-2022 risk categories. **F** Overall survival stratified by ELN-2017 risk categories. **G** Cumulative incidence of relapse stratified by ELN-2022 risk categories. **H** Cumulative incidence of relapse stratified by ELN-2017 risk categories

We reclassified 600 AML patients who underwent allo-HSCT by ELN-2022 genetic risk categories: 214 (35.67%) were favorable-risk, 162 (27.0%) were intermediate-risk and 224 (37.33%) were adverse-risk. Eighty-six (14.33%) patients shifted from ELN-2017 risk stratification (Fig. 1A, B). Reasons for these shifts are detailed in Additional file 1: Table S1.

We assessed the frequency of genetic abnormalities defined by ELN-2022 and the distribution of additional genes mutated in more than 10 patients (Fig. 1C, D). Correlation analysis showed that t(8;21) strongly correlated with KIT mutation (r = 0.5, P < 0.001), SF3B1 mutation strongly correlated with inv(3) (r = 0.5, P < 0.001).

Patients and transplant-related characteristics were listed in Additional file 1: Table S2. Compared to favorable- and intermediate-risk groups, adverse-risk group had a lower percentage of bone-marrow blasts at initial diagnosis (P = 0.036) and a higher proportion of refractory/relapse- and secondary-AML (P = 0.006, p < 0.001, respectively, Additional file 1: Fig. S1).

The three-year and five-year overall survival (OS), event-free survival, cumulative incidence of relapse (CIR) and non-relapse mortality stratified by ELN-2022 and ELN-2017 are shown in Additional file 1: Table S3. Compared to favorable-risk, OS shortened significantly as the ELN-2022 risk stratification increased but didn’t significantly in ELN-2017 intermediate-risk (Fig. 1E, F). Pairwise comparisons for OS revealed significant differences between the ELN-2022 favorable- and intermediate-risk groups (P = 0.047) but not between the intermediate- and adverse-risk groups (P = 0.455). Based on ELN-2017 risk stratification, OS was not significantly different between intermediate- and favorable-risk groups (P = 0.115) or between intermediate- and adverse-risk groups (P = 0.115). Both ELN-2022 and ELN-2017 adverse-risk were associated with increased CIR. (Fig. 1G, H) Smoothed hazard estimates showed a higher mortality risk within 6 months post-transplantation in ELN-2022 intermediate-risk group than in adverse-risk group. Assessment based on ELN-2017 recommendations indicated that adverse-risk group had the highest hazard ratio for death in 1-year post-transplantation, followed by intermediate- and favorable-risk groups (Additional file 1: Fig. S2).

**Fig. 2:**
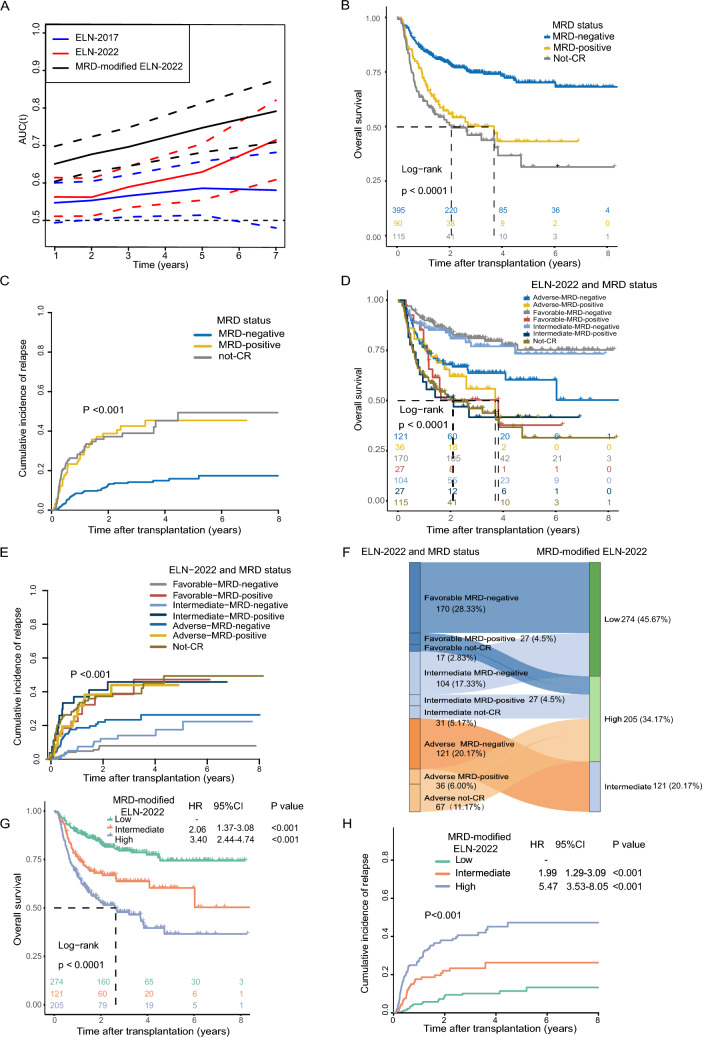
Impact of MRD pre-transplantation and MRD-modified ELN-2022 risk system on clinical outcomes. **A** Dynastic AUC for overall survival at different time points after transplantation according to ELN-2017, ELN-2022 and MRD-modified ELN-2022 risk system. **B** Impact of MRD pre-transplantation on overall survival. **C** Impact of MRD pre-transplantation on cumulative incidence of relapse. **D** Impact of MRD pre-transplantation combined with ELN-2022 risk stratification on overall survival. **E** Impact of MRD pre-transplantation combined with ELN-2022 risk stratification on cumulative incidence of relapse. **F** Relationship of risk groups between ELN-2022 and MRD-modified ELN-2022 risk groups. **G** Overall survival according to MRD-modified ELN-2022 risk groups. **H** Cumulative incidence of relapse according to MRD-modified ELN-2022 risk system

We performed time-dependent receiver operating characteristic (ROC) analysis to validate the prognostic efficacy of ELN-2022 and ELN-2017 risk systems in our transplant cohort. The AUC for predicting OS gradually increased from one to five years post-transplantation, with the AUC for ELN-2022 consistently higher than of ELN-2017 (Fig. 2A). However, AUC for 3-year and 5-year OS between two ELN versions was not significantly different (P = 0.458, P = 0.838, respectively).

We separated patients into three groups based on pre-transplant minimal residual disease (MRD): MRD-negative (395, 65.8%), MRD-positive (90, 15.0%) and not-CR (115, 19.2%). Median survival was not reached for MRD-negative, 3.70 (95% CI 1.6 to NA) years for MRD-positive and 2.07 (95% CI 1.35 to 4.73) years for not-CR patients (P < 0.001) (Fig. 2B, C). Further stratification based on both MRD and ELN-2022 was conducted. The survival of MRD-negative patients in favorable- and intermediate-risk groups was comparable and longer than adverse-risk group. OS and CIR of MRD-positive patients were not significantly different among the three ELN-2022 groups and were similar to Not-CR patients (Fig. 2D, E). Based on aforementioned analysis, we created the MRD-modified ELN-2022 risk system for transplant AML patients. Number and risk-shift of patients from ELN-2022 risk groups to MRD-modified risk groups are shown in Fig. 2F. Three-year OS after transplantation of modified low-, intermediate- and high-risk was 79.5% (95% CI 74.4% to 84.9%), 63.69% (95% CI 55.01% to 73.74%), 47.77% (95% CI 40.79% to 55.94%) (P < 0.001) and 3-year CIR after transplantation was 10.09% (95% CI 6.53% to 14.55%), 23.76 (95% CI 15.82% to 31.79%), 40.65 (95% CI 33.28% to 47.88%) (P < 0.001, Fig. 2G, H). Time-dependent ROC analysis for 3-year survival significantly outperforms ELN-2022 (68.23% vs 53.31%, P < 0.001), as well as for 5-year survival (72.81% vs 58.80%, P < 0.001, Fig. 2A).

In conclusion, ELN-2022 risk system had superior separation for survival of favorable- and unfavorable-risk groups but poor separate for intermediate- and adverse-risk groups. ELN-2017 risk system primarily separates survival of favorable- and adverse-risk groups. Both ELN-2022 and ELN-2017 systems exhibited limited prognostic utility for AML patients undergoing allo-HSCT. Pre-transplant MRD provides additional prognostic insights and MRD-modified ELN-2022 risk system enhances prognostic ability for transplantation.

### Supplementary Information

**Additional file 1.** Additional Patients and Methods; Additional Figures; Additional Tables; Additional References.

## Data Availability

The data that supports the findings is not publicly available for privacy or ethical restrictions. The data are available upon reasonable request from the corresponding author.

## Abbreviations

ELN: European LeukemiaNet
Allo-HSCT: Allogeneic hematopoietic stem cell transplantation
AML: Acute myeloid leukemia
MRD: Minimal residual disease
CR: Complete response
OS: Overall survival
EFS: Event-free survival
CIR: Cumulative incidence of relapse
ROC: Receiver operating characteristic
AUC: The area under the receiver operating characteristic curve
