# Supplementary material for: Outcomes of acute myeloid leukemia patients undergoing allogeneic hematopoietic stem cell transplantation: validation, comparison and improvement of 2022 ELN genetic risk system
Source: Exp Hematol Oncol. 2024 Feb 15;13:16. doi: 10.1186/s40164-024-00487-6 (PMC10870651; doi:10.1186/s40164-024-00487-6)
Supplement: Supplementary file 1 — Additional file 1. Additional Patients and Methods; Additional Figures; Additional Tables; Additional References. [file 40164_2024_487_MOESM1_ESM.docx]

**Outcomes of Acute Myeloid Leukemia Patients Undergoing Allogeneic Hematopoietic Stem Cell Transplantation: Validation, Comparison and Improvement of 2022 ELN Genetic Risk System**

Haixiao Zhang^1,2^#, Xinhui Zheng^1,2^, Yonghui Xia^1^, Rongli Zhang^1,2^, Weihua Zhai^1,2^, Xin Chen^1,2^, Qiaoling Ma^1,2^, Donglin Yang^1,2^, Jialin Wei^1,2^, Aiming Pang^1,2^, Yi He^1,2^, Sizhou Feng^1,2^, Jianxiang Wang^1^, Mingzhe Han^1,2^, Erlie Jiang^1,2^*

^1^State Key Laboratory of Experimental Hematology, National Clinical Research Center for Blood Diseases, Haihe Laboratory of Cell Ecosystem, Institute of Hematology & Blood Diseases Hospital, Chinese Academy of Medical Sciences & Peking Union Medical College, Tianjin, China

^2^Hematopoietic Stem Cell Transplantation Center, Institute of Hematology & Blood Diseases Hospital, Chinese Academy of Medical Sciences & Peking Union Medical College, Tianjin, China

*Corresponding author.

E-mail: [doctor_eljiang@163.com](mailto:doctor_eljiang@163.com) (Erlie Jiang)

**Supplementary Files Content**

Patients and Methods

Table1: Processing of unclear risk stratification for ELN-2022 recommendation.

Table2: Hotspots or protein-coding regions of 141 genes closely associated with hematological disorders.

Table3: Protein-coding regions of 175 genes closely related to hematological disorders.

Table4: Protein-coding regions of 267 genes closely related to hematological disorders.

Table5: A panel of 43 fusion genes.

Table S1: Detailed reasons causing shifts from ELN-2017 to ELN-2022 genetic risk recommendations.

Table S2: Patients and transplantation-related characteristics stratified by ELN-2022 genetic risk system.

Table S3: Survival and relapse of 600 AML patients three and five years after allo-HSCT.

Figure S1: The percentage of blasts in bone marrow and the number of chemotherapy cycles before transplantation distributed in ELN-2022 genetic risk groups.

Figure S2: Estimates of dynamic Hazard Risk for death after transplantation stratified by ELN-2022 and ELN-2017 genetic risk categories.

Reference

**Patients and Methods**

**Study Cohort**

We enrolled 764 consecutive acute myeloid leukemia (AML) patients, diagnosed by 2016 WHO criteria ^[1]^, who underwent allogeneic stem cell transplantation at the Institute of Hematology and Blood Diseases Hospital, Peking Union Medical College, and the Chinese Academy of Medical Sciences (IHCAMS) from January 2014 until December 2021. All cases were included in National Longitudinal Cohort of Hematological Diseases (NICHE, NCT04645199). Cases of Acute Promyelocytic Leukemia (n=3), with autologous hematopoietic stem cell transplantation (n=54), and without genetics results at initial diagnosis (n=107), as defined by ELN-2022, were excluded from this study. Finally, 600 patients were retrospectively analyzed in our study and of whom 487 were reported previously in the “SKIRT” dataset ^[2]^. The diagnostic procedure followed a multidisciplinary process ^[3]^. Risk stratifications of ELN-2017 and ELN-2022 were classified independently by at least two researchers according to genetic characteristics acquired at diagnosis. Processing of unclear risk stratification were listed in **Table 1**. The final follow-up date was October 20, 2022, with median and maximum follow-up time were 2.89 (95% CI 2.67-3.03) years and 8.78 years, respectively. All patients provided informed consent for clinical information for scientific research at the time of first admission. The research protocol was approved by the Ethics Committee of IHCAMS and followed the principles of the Declaration of Helsinki.

**Genetic Studies**

Protocol for karyotype examinations and targeted next-generation sequencing (NGS) was described preciously ^[4]^. In brief, fresh bone marrow (BM) specimens were short-time cultured without mitogen stimulation and conventional chromosome analysis was performed. Karyotype descriptions followed the International System for Human Cytogenetic Nomenclature (2013) recommendations ^[5]^. Genomic DNA was extracted from BM specimens for NGS targeting at least 141 genes involved point mutations and insertion/deletion mutations in protein-coding regions or hotspots. The genes contained in the three NGS panels were seen in **Table 2-4**. FLT3-ITD mutation was validated by polymerase chain reaction and capillary electrophoresis. Validation of NPM1-exon12, CEBPA-TAD, CEBPA-bZIP, carl-exon9, and MPL-exon10 was performed by using Sanger sequencing. Fusion genes were detected using a panel comprising 43 targets (**Table 5**). Total RNA was extracted from BM specimens, reverse transcribed to cDNA, and the expression of fusion genes in the transcriptome was qualitatively analyzed.

**Multiparameter flow cytometry-based MRD detection**

Minimal residual disease (MRD) of BM specimens was monitored by multiparameter flow cytometer (MFC) with a sensitivity level of 10-4 and any level of measurable MRD was defined as MRD-positive and conversely was defined as MRD-negative. Concretely, a volume of 3-5 ml fresh BM samples was collected in heparinized tubes and was detected by CD45/SSC-gated eight-colors flow cytometry within 24 hours. The panel used for AML MRD detection includes the following two tubes: (1) CD38-FITC/CD117-PE/CD34PE-Cy5/CD33-PE-Cy5/CD13-APC/HLA-DR-APC-H7/CD11b-BV450/CD45-BV500, CD15-FITC/CD34-PE/CD56-PE-Cy5/CD33-PE-C7/CD7-APC/CD14-APC-H7/CD19-BV450/CD45-BV500. All monoclonal antibodies were obtained from BD, BECKMAN or Biolegend. Samples were detected by BD FACS Canto flow cytometer. Five hundred thousand nucleated cells were analyzed at least and data analysis were conducted using software Kaluza Analysis1.3. Leukemia-associated immunophenotype (LAIP) defined at AML diagnosis was used for MFC-MRD detection. The different-from-normal immunophenotype was performed to monitor MFC-*MRD* when *LAIP* was not available at diagnosis. Pre-transplant MRD was obtained within fifteen days before conditioning.

**Procedure of Allo-HSCT**

Chemotherapy regimens were administered to patients according to protocols at IHCAMS ^[6]^. The International Working Group criteria were followed to define clinical responses ^[7]^, and complete response (CR) was defined as morphologic leukemia-free state. At least 10 HLA loci (HLA-A, B, DRB1, C and DQ) were tested, and priority was given to HLA-matched sibling donors (MSD). All patients received modified myeloablative conditioning regimens based on modified busulfan and cyclophosphamide with the addition of anti-thymocyte globulin for matched unrelated donors (MUD), mismatched related donors (MMRD) and mismatched unrelated donors (MMUD) transplantation. Acute graft versus host disease (GvHD) was diagnosed and graded according to the MAGIC criteria ^[7]^. Cyclosporine A or tacrolimus in combination with a short course of methotrexate with or without mycophenolate mofetil were used to prevent acute GvHD. Methylprednisolone was used as first-line treatment for acute GvHD. Neutrophil engraftment was defined as the first day of three consecutive days with a count of neutrophils more than 0.5×10 9 /L in peripheral blood. [Platelet](javascript:;) engraftment was defined as the first day of seven consecutive days with a count of platelets more than 20×10 9 /L in peripheral blood, without transfusion. BM monitoring for disease status during chemotherapy and allo-HSCT period at various time points was in agreement with the principles summarized by Percival et al ^[8]^.

**Statistical Analyses**

We conducted data analysis using R 4.1.2 and GraphPad Prism 8.0.2 software. The Mann-Whitney test was used for continuous data and the Chi-square test or Fisher's exact test was used for categorized data. Pearson correlation analysis was used to calculate correlations between variables. Kaplan-Meier method was used for overall survival (OS) and event-free survival (EFS) calculation from transplantation until death and relapse/death, respectively, and the Log-rank test was used for comparison between groups. Median follow-up time was calculated using the Reverse Kaplan-Meier method. Cumulative incidence of relapse (CIR) and non-relapse mortality (NRM) were analyzed using competitive analysis, and group comparisons were made using Gray's test ^[9]^. Cox proportional hazards regression and Fine-Gray competing risks regression models were used to define hazard ratios (HRs). Smoothed hazard estimates were calculated based on “RPEXE” ^[10]^. The time-dependent receiver operating characteristic (ROC) curve and the area under the receiver operating characteristic curve (AUC) at various time points after allo-HSCT were used to evaluate the prognostic efficacy of 2022, 2017 and MRD-modified ELN risk systems. A two-sided test of P < 0.05 was defined as the threshold for statistical significance in all analyses.

**Table1: Processing of unclear risk stratification for ELN-2022 recommendation.**

| Characteristics | Combined with characteristics | Number of patients | Finally risk stratification |
| --- | --- | --- | --- |
| Biallelic mutated CEBPA/bZIP in-frame mutated CEBPA | Mutated FLT3-ITD ^[11]^ | 8 | favorable |
|  | t(v;11q23.3)/KMT2A-rearranged | 1 | adverse |
|  | Mutated TP53 | 1 | adverse |
|  | -5 or del(5q) | 1 | adverse |
| t(8;21) | Mutated FLT3-ITD | 5 | favorable |
|  | -5 or del(5q) | 1 | adverse |
|  | t(v;11q23.3)/KMT2A-rearranged | 1 | adverse |
|  | Mutated ASXL1; Mutated TP53; Mutated FLT3-ITD | 1 | adverse |
|  | Mutated ASXL1; Mutated TP53 | 1 | adverse |
| inv(16) | FLT3-ITD ^[11]^ | 2 | favorable |
|  | Mutated TP53 | 1 | adverse |
|  | −5 or del(5q); −17/abn(17p) | 2 | adverse |

**Table2:** **Hotspots or protein-coding regions of 141 genes closely associated with hematological disorders.**

| gene | sequenced regions | gene | sequenced regions | gene | sequenced regions | gene | sequenced regions |
| --- | --- | --- | --- | --- | --- | --- | --- |
| ABL1 | CDS | CSNK1A1 | Exon2-4 | KLF2 | Exon1-3 | RUNX1 | CDS |
| ANKBD26 | Exon1/5’UTR | CUX1 | CDS | KMT2A | CDS | SETBP1 | CDS |
| ARID1A | CDS | CXCR4 | CDS | KMT2D | CDS | SETD2 | CDS |
| ASXL1 | CDS | DDX3X | CDS | KRAS | CDS | SF1 | CDS |
| ASXL2 | CDS | DDX41 | CDS | MAP2K1 | Exon2-3 | SF3B1 | CDS |
| ATG2B | CDS | DIS3 | CDS | MAPK1 | CDS | SH2B3 | CDS |
| ATM | CDS | DNM2 | Exon8/13/16/18/20 | MAX | CDS | SMC1A | CDS |
| B2M | CDS | DNMT3A | CDS | MED12 | CDS | SMC3 | CDS |
| BCL2 | Exon2 | DNMT3B | CDS | MEF2B | Exon2-3 | SPEN | Exon11 |
| BCL6 | 5'UTR | EED | CDS | MPL | CDS | SRP72 | CDS |
| BCOR | CDS | EGR1 | CDS | MYC | CDS | SRSF2 | CDS |
| BCORL1 | CDS | EP 300 | CDS | MYD88 | CDS | STAG2 | CDS |
| BIRC3 | CDS | ETNK1 | CDS | NF1 | CDS | STAT3 | CDS |
| BRAF | CDS | ETV6 | CDS | NOTCH1 | CDS | STAT5B | Exon11/13-18 |
| BRINP3 | CDS | EZH2 | CDS | NOTCH2 | CDS | SUZ12 | CDS |
| BTK | Exon5/11/14-19 | FAM46C | CDS | NPM1 | CDS | TAL1 | Exon3 |
| CALR | CDS | FAT1 | CDS | NRAS | CDS | TCF3 | Exon6/15/17 |
| CARD11 | CDS | FBXW7 | CDS | NT5C2 | Exon9-16 | TERT | CDS |
| CASP8 | Exon10 | FGFR3 | CDS | PAX5 | CDS | TET2 | CDS |
| CBL | CDS | FLT3 | CDS | PDGFRB | Exon18 | TNFAIP3 | CDS |
| CCND1 | CDS | GATA1 | CDS | PH6 | CDS | TNFRSF14 | Exon1-6 |
| CCND2 | Exon4 | GATA2 | CDS | PIGA | CDS | TP53 | CDS |
| CCND3 | CDS | GATA3 | CDS | PLCG1 | CDS | TPMT | CDS |
| CCR4 | CDS | GNA13 | Exon1-4 | PLCG2 | CDS | TRAF3 | CDS |
| CD28 | Exon4 | ID3 | CDS | PPM1D | CDS | U2AF1 | CDS |
| CD58 | Exon2-3 | IDH1 | CDS | PRDM1 | CDS | USP7 | CDS |
| CD79B | CDS | IDH2 | CDS | PRKCB | CDS | WHSC1 | CDS |
| CDC25C | Exon8 | IKZK1 | CDS | PRPS1 | CDS | WT1 | CDS |
| CDKN1B | CDS | IL7R | Exon5-6 | PTEN | CDS | XPO1 | CDS |
| CDKN2A | CDS | IRF4 | CDS | PTPN11 | CDS | ZBTB7A | CDS |
| XEBPA | CDS | JAK1 | CDS | RAD21 | CDS | ZMYM | CDS |
| CNOT3 | Exon2-5 | JAK2 | CDS | RBBP6 | CDS | ZRSR2 | CDS |
| CREBBP | CDS | JAK3 | CDS | RELN | CDS | PRPF3 | CDS |
| CRLF2 | Exon6 | KDM6A | CDS | RHOA | Exon2-5 | NOTCH3 | CDS |
| CSF3R | CDS | KIT | CDS | RPL10 | Exon5 | NOTCH4 | CDS |
| ZNF384 | CDS |  |  |  |  |  |  |

**Table3: Protein-coding regions of 175 genes closely related to hematological disorders.**

| ABCB1 | ABL1 | ANKBD26 | APC | ARID1A | ARID1B | ARID2 | ASXL1 | ATG2B | ATM |
| --- | --- | --- | --- | --- | --- | --- | --- | --- | --- |
| ATRX | B2M | BCL10 | BCL2 | BCL6 | BCOR | BCORL1 | BIRC3 | BLM | BPGM |
| BRAF | BRCA1 | BRCA2 | BRIP1 | BTG1 | BTK | CALR | CARD11 | CBL | CBLB |
| CBLC | CCND1 | CCND3 | CD28 | CD58 | CD79A | CD79B | CDKN1A | CDKN2A | CDKN2B |
| CEBPA | CHD8 | CIITA | CREBBP | CRLF2 | CSF1R | CSF3R | CTCF | CUX1 | CXCR4 |
| DDX41 | DIS3 | DKC1 | DNM2 | DNMT3A | EED | EGFR | EGLN1 | ELANE | EP300 |
| EPHA7 | EPOR | ETV6 | EZH2 | FAM46C | FAS | FAT1 | FBX011 | FBXW7 | FLT3 |
| FOX01 | GATA1 | GATA2 | CFI1 | GNA13 | GNAI2 | GNAS | GNB1 | GSKIP | HAX1 |
| HRAS | ID3 | IDH1 | IDH2 | IKZF1 | IKZF2 | IKZF3 | IL7R | IRF4 | IRF8 |
| ITPKB | JAK1 | JAK2 | JAK3 | KDM6A | KIT | KMT2A | KMT2B | KMT2C | KMT2D |
| KRAS | KRT20 | LMO2 | LYN | MAP2K1 | MCL1 | MEF2B | MFHAS1 | MPL | MTOR |
| MYC | MYD88 | MYOM2 | NF1 | NOTCH1 | NOTCH2 | NPM1 | NRAS | NT5C2 | PALB2 |
| PAX5 | PDGFRA | PDGFRB | PHF6 | PIGA | PIK3CA | PIK3CD | PIM1 | PLCG2 | PPM1D |
| PRDM1 | PRF1 | PRKDC | PRPF8 | PTEN | PTPN11 | RAD21 | RELN | RHOA | RUNX1 |
| SBDS | SETBP1 | SETD2 | SETDB1 | SFSB1 | SGK1 | SH2B3 | SWC1A | SMC3 | SOCS1 |
| SRP72 | SRSF2 | STAG2 | STAT3 | STAT5B | STAT6 | SUZ12 | SYK | TAL1 | TCF3 |
| TERC | TERT | TET2 | TNFAIP3 | TNFRSF14 | TP53 | TPMT | TRAF3 | U2AF1 | VHL |
| WHSC1 | WT1 | XPO1 | ZAP70 | ZRSR2 |  |  |  |  |  |

**Table4: Protein-coding regions of 267 genes closely related to hematological disorders.**

| ABCB1 | ABL1 | ANKBD26 | APC | ARID1A | ARID1B | ARID2 | ARID5B | ASXL1 | ASXL2 |
| --- | --- | --- | --- | --- | --- | --- | --- | --- | --- |
| ATG2B | ATM | ATRX | B2M | BACH2 | BCL10 | BCL2 | BCL6 | BCL7A | BCOR |
| BCORL1 | BIRC3 | BLM | BPGM | BRAF | BRCA1 | BRCA2 | BRIP1 | BTG1 | BTG2 |
| BTK | CALR | CARD11 | CBL | CBLB | CBLC | CCND1 | CCND3 | CCR4 | CD28 |
| CD58 | CD79A | CD79B | CDC25C | CDKN1A | CDKN1B | CDKN2A | CDKN2B | CDKN2C | CEBPA |
| CHD2 | CHD8 | CIITA | CNOT3 | CREBBP | CRLF2 | CSF1R | CSF3R | CSMD1 | CSNK1A1 |
| CTCF | CUX1 | CXCR4 | CYLD | DDX3X | DDX41 | DIS3 | DKC1 | DNM2 | DNMT3A |
| DNMT3B | DTX1 | DUSP2 | EBF1 | EED | EGFR | EGLN1 | EGR1 | ELANE | EP300 |
| EPHA7 | EPOR | ETNK1 | ETV6 | EZH2 | FAM46C | FAS | FAT1 | FAT4 | FBX011 |
| FBXW7 | FGFR3 | FLT3 | FOX01 | FYN | GAB2 | GATA1 | GATA2 | GATA3 | CFI1 |
| GNA13 | GNAI2 | GNAS | GNB1 | GSKIP | H1-2 | H1-3 | H1-4 | H1-5 | HAX1 |
| HLA-A | HLA-C | HLA-DMB | HNRNPK | HRAS | HUWE1 | HVCN1 | ID3 | IDH1 | IDH2 |
| IGLL5 | IKZF1 | IKZF2 | IKZF3 | IL7R | IRF2BP2 | IRF4 | IRF8 | ITPKB | JAK1 |
| JAK2 | JAK3 | JUNB | KDM6A | KIT | KLF2 | KLH6 | KMT2A | KMT2B | KMT2C |
| KMT2D | KRAS | KRT20 | LCOR | LMO2 | LTB | LYN | MAP2K1 | MAPK1 | MAX |
| MCL1 | MED12 | MEF2B | MFHAS1 | MPL | MTOR | MYC | MYCN | MYD88 | MYOM2 |
| NF1 | NFE2 | NFKBIA | NFKBIE | NOTCH1 | NOTCH2 | NOTCH3 | NOTCH4 | NPM1 | NRAS |
| NT5C2 | P2RY8 | PALB2 | PAX5 | PDGFRA | PDGFRB | PDS5B | PHF6 | PIGA | PIK3CA |
| PIK3CD | PIK3R1 | PIM1 | PIM2 | PLCG1 | PLCG2 | POT1 | PPM1D | PRDM1 | PRF1 |
| PRKCB | PRKD2 | PRKDC | PRPF8 | PRPS1 | PSMB5 | PTEN | PTPN1 | PTPN11 | PTPRD |
| RAD21 | RASA2 | RB1 | RBBP6 | RELN | RHOA | RPL10 | RRAGC | RUNX1 | SAMHD1 |
| SBDS | SETBP1 | SETD1B | SETD2 | SETDB1 | SF1 | SFSB1 | SGK1 | SH2B3 | SH2D1A |
| SMARCA4 | SWC1A | SMC3 | SMO | SOCS1 | SP140 | SPEN | SRP72 | SRSF2 | STAG2 |
| STAT3 | STAT5B | STAT6 | SUFU | SUZ12 | SYK | TAL1 | TBL1XR1 | TCF3 | TERC |
| TERT | TET1 | TET2 | TMEM30A | TMSB4X | TNFAIP3 | TNFRSF14 | TOX | TP53 | TPMT |
| TRAF3 | U2AF1 | UBE2A | UBR5 | USP7 | VAV1 | VHL | WHSC1 | WT1 | XBP1 |
| XPO1 | ZAP70 | ZBTB7A | ZFP36L1 | ZMYM3 | ZNF292 | ZRSR2 |  |  |  |

**Table5: A panel of 43 fusion genes.**

| BCR-ABL | AML1-ETO | MLL-AF9 | MLL-ENL |
| --- | --- | --- | --- |
| MLL-SEPT6 | MLL-AF17 | MLL-AF1p | NPM-RARA |
| AML1-MDS1/EV11 | TEL-ABL | TEL-AML1 | E2A-PBX1 |
| SIL-TAL1 | DEK-CAN | STAT5b-RARA | NUP98-HOXA13 |
| NUP98-HOXD13 | MLL-AFX | PRKAR1A-RARA | NPM-ALK |
| TLS-ERG | PML-RARA(L) | CBFβ-MYH11 | MLL-AF4 |
| MLL-AF10 | MLL-ELL | MLL-AF1q | MLL-AF6 |
| PLZF-RARA | AML1-MTG16 | TEL-JAK2 | TEL-PDGFRB |
| E2A-HLF | FIP1L1-PDGFRA | NPM-MLF1 | ETV6-PDGFRA |
| NUP98-HOXC11 | NUP98-HOXA9 | NUP98-PMX1 | FIP1L1-RARA |
| NUMA1-RARA | SET-CAN | WT1 (expression) |  |

**TableS1:** **Detailed reasons causing shifts from ELN-2017 to ELN-2022 genetic risk recommendations**

| ELN-2017 | | ELN-2022 | | number of patients |
| --- | --- | --- | --- | --- |
| Favorable | Mutated NPM1 without FLT3-ITD or with FLT3-ITDlow | Mutated NPM1 with FLT3-ITD | Intermediate | 8 |
|  | Wild-type NPM1 without FLT3-ITD or with FLT3-ITDlow; Biallelic mutated CEBPA | Wild-type NPM1 with FLT3-ITD |  | 1 |
|  | Biallelic mutated CEBPA | Cytogenetic and/or molecular abnormalities not classified as favorable or adverse |  | 3 |
|  | Biallelic mutated CEBPA; Mutated NPM1 and FLT3-ITDhigh | Mutated NPM1 with FLT3-ITD |  | 1 |
|  | Mutated ASXL1; Biallelic mutated CEBPA | Mutated ASXL1 | Adverse | 1 |
| Intermediate | Cytogenetic abnormalities not classified as favorable or adverse | bZIP in-frame mutated CEBPA | Favorable | 9 |
|  | Wild-type NPM1 without FLT3-ITD or with FLT3-ITDlow | Wild-type NPM1 with FLT3-ITD; bZIP in-frame mutated CEBPA |  | 1 |
|  | Cytogenetic abnormalities not classified as favorable or adverse | Mutated BCOR, EZH2, RUNX1, SF3B1, SRSF2, STAG2, U2AF1, or ZRSR2 | Adverse | 39 |
|  | Cytogenetic abnormalities not classified as favorable or adverse | t(3q26.2;v)/MECOM(EVI1)-rearranged |  | 1 |
|  | Cytogenetic abnormalities not classified as favorable or adverse | t(8;16)(p11;p13)/KAT6A::CREBBP;Mutated ZRSR2 |  | 1 |
|  | Wild-type NPM1 without FLT3-ITD or with FLT3-ITDlow | Wild-type NPM1 with FLT3-ITD；Mutated U2AF1/STAG2 |  | 2 |
| Adverse | Wild-type NPM1 and FLT3-ITDhigh | bZIP in-frame mutated CEBPA; Wild-type NPM1 with FLT3-ITD | favorable | 1 |
|  | t(8;21)(q22;q22.1); RUNX1-RUNX1T1; Mutated TP53(VAF<10%) | t(8;21)(q22;q22.1); RUNX1-RUNX1T1 |  | 1 |
|  | MLL-PTD | Cytogenetic and/or molecular abnormalities not classified as favorable or adverse | Intermediate | 3 |
|  | Wild-type NPM1 and FLT3-ITDhigh | Wild-type NPM1 with FLT3-ITD |  | 13 |
|  | Mutated TP53(VAF<10%) | Cytogenetic and/or molecular abnormalities not classified as favorable or adverse |  | 1 |

**TableS2: Patients and transplantation-related characteristics stratified by ELN-2022 genetic risk system.**

|  | Total | Favorable | Intermediate | Adverse | p |
| --- | --- | --- | --- | --- | --- |
| n | 600 | 214 | 162 | 224 |  |
| **Patient Characteristic** | | | | | |
| Diagnosis age, years (mean (SD)) | 36.87 (11.81) | 37.42 (11.96) | 35.19 (12.50) | 37.56 (11.07) | 0.106 |
| Patient sex (%) |  |  |  |  | 0.793 |
| Male | 325 (54.2) | 112 (52.3) | 90 (55.6) | 123 (54.9) |  |
| Female | 275 (45.8) | 102 (47.7) | 72 (44.4) | 101 (45.1) |  |
| WBC, 109/L (mean (SD)) | 34.51 (54.05) | 39.31 (58.70) | 33.50 (44.23) | 30.66 (55.74) | 0.237 |
| Hb, g/L (mean (SD)) | 83.83 (24.88) | 83.75 (22.70) | 85.99 (25.87) | 82.33 (26.12) | 0.362 |
| PLT, 109/L (mean (SD)) | 67.30 (70.43) | 58.83 (70.63) | 69.51 (59.93) | 73.80 (76.49) | 0.076 |
| Blasts in BM (mean (SD)) | 56.77 (23.55) | 58.03 (22.02) | 59.43 (24.17) | 53.65 (24.27) | 0.036 |
| Type of FAB (%) |  |  |  |  | <0.001 |
| M0 | 7 (1.2) | 0 (0.0) | 0 (0.0) | 7 (3.1) |  |
| M1 | 14 (2.3) | 9 (4.2) | 3 (1.9) | 2 (0.9) |  |
| M2 | 148 (24.7) | 104 (48.6) | 23 (14.2) | 21 (9.4) |  |
| M4 | 63 (10.5) | 37 (17.3) | 15 (9.3) | 11 (4.9) |  |
| M5 | 288 (48.0) | 53 (24.8) | 94 (58.0) | 141 (62.9) |  |
| M6 | 7 (1.2) | 1 (0.5) | 4 (2.5) | 2 (0.9) |  |
| M7 | 1 (0.2) | 0 (0.0) | 1 (0.6) | 0 (0.0) |  |
| Missing | 72 (12.0) | 10 (4.7) | 22 (13.6) | 40 (17.9) |  |
| Extramedullary infiltration (%) |  |  |  |  | 0.523 |
| No | 550 (91.7) | 197 (92.1) | 148 (91.4) | 205 (91.5) |  |
| CNS | 40 (6.7) | 15 (7.0) | 9 (5.6) | 16 (7.1) |  |
| Not CNS | 10 (1.7) | 2 (0.9) | 5 (3.1) | 3 (1.3) |  |
| Primary or secondary AML (%) |  |  |  |  | <0.001 |
| primary AML | 535 (89.2) | 210 (98.1) | 148 (91.4) | 177 (79.0) |  |
| secondary AML | 65 (10.8) | 4 (1.9) | 14 (8.6) | 47 (21.0) |  |
| ELN-2017 risk group (%) |  |  |  |  | <0.001 |
| Favorable | 216 (36.0) | 202 (94.4) | 13 (8.0) | 1 (0.4) |  |
| Intermediate | 185 (30.8) | 10 (4.7) | 132 (81.5) | 43 (19.2) |  |
| Adverse | 199 (33.2) | 2 (0.9) | 17 (10.5) | 180 (80.4) |  |
| Total cycles of chemotherapy (%) |  |  |  |  | <0.001 |
| 0 | 15 (2.5) | 0 (0.0) | 2 (1.2) | 13 (5.8) |  |
| 1 to 4 | 403 (67.2) | 123 (57.5) | 114 (70.4) | 166 (74.1) |  |
| >5 | 182 (30.3) | 91 (42.5) | 46 (28.4) | 45 (20.1) |  |
| Relapse/refractory AML (%) |  |  |  |  | 0.006 |
| Yes | 252 (42.0) | 74 (34.6) | 67 (41.4) | 111 (49.6) |  |
| No | 448 (58.0) | 140 (65.4) | 95 (58.6) | 113 (50.4) |  |
| **Transplantation-related Characteristics** | | | | | |
| Year of SCT (%) |  |  |  |  | 0.219 |
| 2014 to 2017 | 136 (22.7) | 56 (26.2) | 37 (22.8) | 43 (19.2) |  |
| 2018 to 2021 | 464 (77.3) | 158 (73.8) | 125 (77.2) | 181 (80.8) |  |
| SCT age, years (mean (SD)) | 37.61 (11.88) | 38.38 (12.06) | 35.90 (12.56) | 38.12 (11.10) | 0.096 |
| Time to SCT, days (mean (SD)) | 279.02 (241.02) | 342.08 (282.22) | 271.28 (253.84) | 224.37 (163.04) | <0.001 |
| Status pre-SCT (%) |  |  |  |  | <0.001 |
| CR1 | 441 (73.5) | 169 (79.0) | 121 (74.7) | 151 (67.4) |  |
| CR2 | 44 (7.3) | 28 (13.1) | 10 (6.2) | 6 (2.7) |  |
| PR | 8 (1.3) | 1 (0.5) | 2 (1.2) | 5 (2.2) |  |
| NR | 32 (5.3) | 0 (0.0) | 8 (4.9) | 24 (10.7) |  |
| Relapse | 60 (10.0) | 16 (7.5) | 19 (11.7) | 25 (11.2) |  |
| Upfront | 15 (2.5) | 0 (0.0) | 2 (1.2) | 13 (5.8) |  |
| MRD status pre-SCT (%) |  |  |  |  | <0.001 |
| Negative | 395 (65.8) | 170 (79.4) | 104 (64.2) | 121 (54.0) |  |
| Positive | 90 (15.0) | 27 (12.6) | 27 (16.7) | 36 (16.1) |  |
| Not-CR | 115 (19.2) | 17 (7.9) | 31 (19.1) | 67 (29.9) |  |
| HCT-CI score (%) |  |  |  |  | 0.641 |
| 0 | 477 (79.5) | 171 (79.9) | 130 (80.2) | 176 (78.6) |  |
| 1-2 scores | 109 (18.2) | 40 (18.7) | 29 (17.9) | 40 (17.9) |  |
| > 2 scores | 14 (2.3) | 3 (1.4) | 3 (1.9) | 8 (3.6) |  |
| SCT type (%) |  |  |  |  | 0.901 |
| MMRD | 331 (51.8) | 107 (50.0) | 88 (54.3) | 116 (51.8) |  |
| MMUD | 6 (1.0) | 2 (0.9) | 1 (0.6) | 3 (1.3) |  |
| MSD | 258 (43.0) | 98 (45.8) | 66 (40.7) | 94 (42.0) |  |
| MUD | 25 (4.2) | 7 (3.3) | 7 (4.3) | 11 (4.9) |  |
| Source of HSC (%) |  |  |  |  | 0.616 |
| BM | 1 (0.2) | 1 (0.5) | 0 (0.0) | 0 (0.0) |  |
| PB | 579 (96.5) | 204 (95.3) | 157 (96.9) | 218 (97.3) |  |
| PB+BM | 20 (3.3) | 9 (4.2) | 5 (3.1) | 6 (2.7) |  |
| Donor age, year (mean (SD)) | 35.78 (13.12) | 35.86 (12.88) | 35.20 (12.80) | 36.12 (13.61) | 0.79 |
| Gender match, donor to patient (%) | |  |  |  | 0.635 |
| Female to Female | 87 (14.5) | 37 (17.3) | 24 (14.8) | 26 (11.6) |  |
| Female to male | 114 (19.0) | 42 (19.6) | 33 (20.4) | 39 (17.4) |  |
| Male to Female | 190 (31.7) | 66 (30.8) | 48 (29.6) | 76 (33.9) |  |
| Male to Male | 209 (34.8) | 69 (32.2) | 57 (35.2) | 83 (37.1) |  |
| GvHD prophylaxis (%) |  |  |  |  | 0.655 |
| CSA+MTX | 125 (20.8) | 42 (19.6) | 35 (21.6) | 48 (21.4) |  |
| CSA+MTX+MMF | 139 (23.2) | 53 (24.8) | 39 (24.1) | 47 (21.0) |  |
| FK506+MTX | 144 (24.0) | 58 (27.1) | 37 (22.8) | 49 (21.9) |  |
| FK506+MTX+MMF | 192 (32.0) | 61 (28.5) | 51 (31.5) | 80 (35.7) |  |
| CD34 + cell, 10 6 cells/kg (mean (SD)) | 3.18 (1.26) | 3.17 (1.24) | 3.16 (1.22) | 3.20 (1.30) | 0.964 |
| ANC engraftment, days (mean (SD)) | 13.42 (3.01) | 13.86 (3.53) | 12.74 (2.33) | 13.50 (2.82) | 0.002 |
| PLT engraftment, days (mean (SD)) | 18.76 (15.47) | 18.99 (12.97) | 18.25 (12.95) | 18.92 (19.00) | 0.883 |
| Maintenance treatment post-SCT (%) |  |  |  |  | 0.153 |
| Yes | 105 (17.5) | 38 (17.8) | 21 (13.0) | 46 (20.5) |  |
| No | 495 (82.5) | 176 (82.2) | 141 (87.0） | 178 (79.5) |  |
| Time of follow-up, years (mean (SD)) | 2.41 (1.83) | 2.73 (1.92) | 2.35 (1.96) | 2.14 (1.61) | 0.003 |
| Acute GvHD (%) |  |  |  |  | 0.457 |
| I° GvHD | 179 (29.8) | 62 (29.0) | 51 (31.5) | 66 (29.5) |  |
| II-IV° GvHD | 94 (15.7) | 27 (12.6) | 30 (18.5) | 37 (16.5) |  |
| No | 327 (54.5) | 125 (58.4) | 81 (50.0) | 121 (54.0) |  |
| Relapse (%) |  |  |  |  | 0.004 |
| Yes | 215 (35.8) | 59 (27.6) | 60 (37.0) | 96 (42.9) |  |
| No | 385 (64.2) | 155 (72.4) | 102 (63.0） | 128 (57.1) |  |

WBC, white blood cells; Hb, hemoglobin; PLT, platelets; BM, bone marrow; FAB, French-American British classification; CNS, central nervous system; AML, acute myeloid leukemia; SCT, stem cell transplant; CR, complete remission; PR, partial remission; NR, no remission; MRD, minimal residual disease; MMRD, mismatched related donors; MMUD, mismatched unrelated donors; MSD, matched sibling donors; MUD, matched unrelated donors; PB, peripheral blood; CSA, cyclosporine A; MTX, methotrexate; MMF, mycophenolate mofetil; FK506, tacrolimus; ANC, absolute neutrophil count; GvHD, graft versus host disease.

**
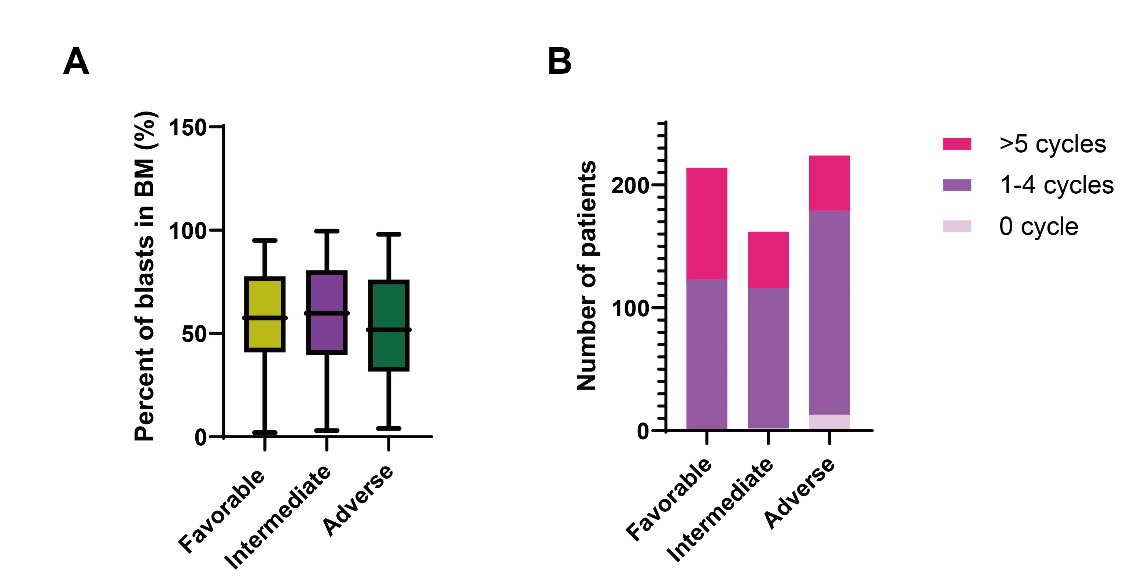
FigureS1:** **The percentage of** **blasts in bone marrow and the number of chemotherapy cycles before transplantation distributed in ELN-2022 genetic risk groups.**

**(A) Percent of blasts in BM. (B) Cycles of chemotherapy before transplantation.**

**Table S3: Survival and relapse of 600 AML patients three and five years after allo-HSCT.**

|  | OS (%,95%CI) | | EFS (%,95%CI) | | CIR (%,95%CI) | | NRM (%,95%CI) | |
| --- | --- | --- | --- | --- | --- | --- | --- | --- |
|  | 3-year | 5-year | 3-year | 5-year | 3-year | 5-year | 3-year | 5-year |
| Favorable | 73.8 (67.8-80.3) | 65.9 (57.8-75.2) | 72.0 (65.9-78.6) | 67.6 (60.4-75.8) | 14.6 (10.1-20.0) | 15.8 (10.8-21.7) | 19.3 (14.0-25.3) | 23.9 (16.9-31.6) |
| Intermediate | 63.9 (56.7-72.0) | 62.6 (55.2-71.0) | 61.5 (54.3-69.6) | 56.7 (48.6-66.2) | 28.6 (20.4-34.2) | 30.5 (22.6-38.7) | 15.8 (10.8-21.6) | 17.1 (11.6-23.3) |
| Adverse | 57.6 (50.4-65.9) | 46.2 (36.4-58.8) | 52.5 (45.2-60.9) | 41.7 (32.4-53.7) | 29.1 (22.3-36.3) | 35.5 (26.2-45.0) | 24.4 (18.1-31.3) | 30.8 (21.8-40.1) |
| Favorable | 73.7 (67.7-80.2) | 67.4 (59.6-76.3) | 71.4 (65.3-78.0) | 66.9 (59.4-75.2) | 15.1 (10.5-20.6) | 16.3 (11.3-22.2) | 19.4 (14.1-25.4) | 24.2 (17.0-32.1) |
| Intermediate | 60.9 (52.9-70.1) | 58.8 (50.3-68.8) | 60.3 (52.4-69.4) | 56.6 (48.0-66.8) | 24.7 (17.7-32.4) | 28.4 (20.1-37.2) | 19.2 (13.1-26.0) | 19.2 (13.1-26.0) |
| Adverse | 60.7 (54.1-68.1) | 49.5 (40.2-60.9) | 55.2 (48.5,62.8) | 44.8 (36.2-55.5) | 30.1 (23.6-36.7) | 35.5 (27.0-44.0) | 20.6 (15.2- 26.6) | 27.2 (19.3, 35.7) |

OS, overall survival; EFS, event-free survival; CIR, cumulative incidence of relapse; NRM cumulative incidence of non-relapse.

**Figure S2: Estimates of dynamic Hazard Risk for death in 600 AML patients with allo-HSCT** **stratified by ELN-2022 and ELN-2017 risk genetic groups.**

**
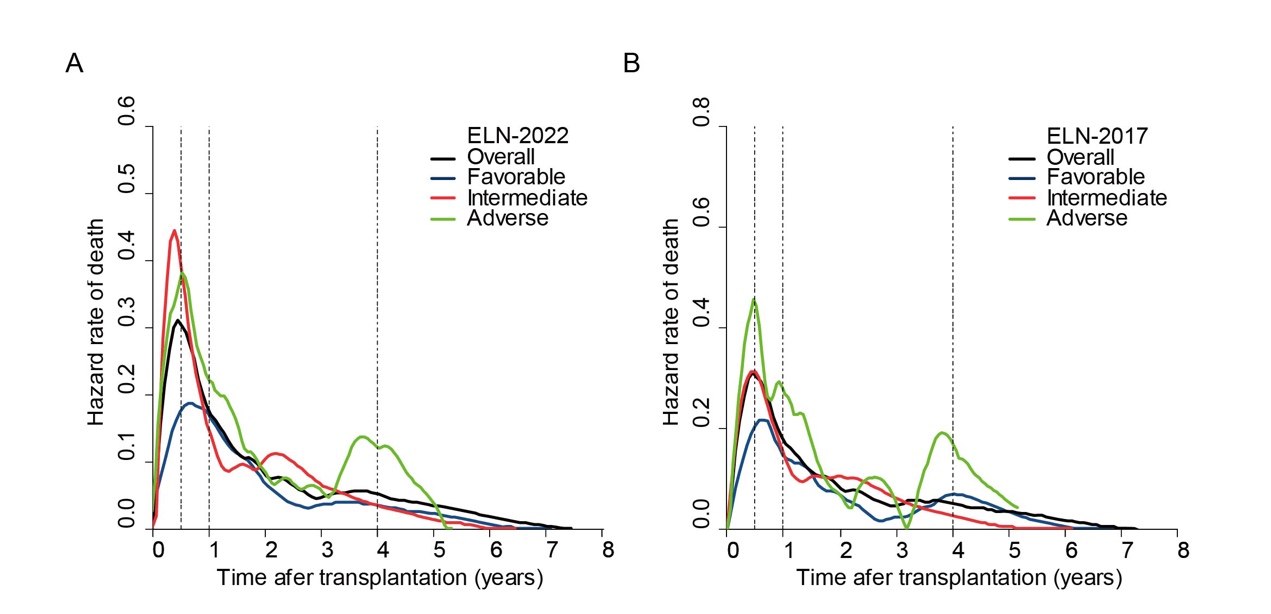
**

(A) Estimates of dynamic Hazard Risk for death stratified by ELN-2022 risk genetic groups. (B) Estimates of dynamic Hazard Risk for death stratified by to ELN-2017 risk genetic groups.

[1]. Arber DA, Orazi A, Hasserjian R, et al. The 2016 revision to the World Health Organization classification of myeloid neoplasms and acute leukemia. *Blood*. May 19 2016;127(20):2391-405. doi:10.1182/blood-2016-03-643544

[2]. Cao Y, Gong X, Feng Y, et al. The Composite Immune Risk Score predicts overall survival after allogeneic hematopoietic stem cell transplantation: A retrospective analysis of 1838 cases. *Am J Hematol*. Feb 2023;98(2):309-321. doi:10.1002/ajh.26792

[3]. Roug AS, Hansen MC, Nederby L, Hokland P. Diagnosing and following adult patients with acute myeloid leukaemia in the genomic age. *Br J Haematol*. Oct 2014;167(2):162-76. doi:10.1111/bjh.13048

[4]. Zhang Y, Wu J, Qin T, et al. Comparison of the revised 4th (2016) and 5th (2022) editions of the World Health Organization classification of myelodysplastic neoplasms. *Leukemia*. Dec 2022;36(12):2875-2882. doi:10.1038/s41375-022-01718-7

[5]. Arber DA, Orazi A, Hasserjian RP, et al. International Consensus Classification of Myeloid Neoplasms and Acute Leukemias: integrating morphologic, clinical, and genomic data. *Blood*. Sep 15 2022;140(11):1200-1228. doi:10.1182/blood.2022015850

[6]. Li Y, Zhang J, Wei H, et al. Clinical, Cytogenetic Characteristics and Survival of Acute Leukemia in a National Research Center Database, 10-Year Real-World Data Review. *Blood*. 2021/11/23/ 2021;138:3062. doi:https://doi.org/10.1182/blood-2021-148412

[7]. Cheson BD, Bennett JM, Kopecky KJ, et al. Revised recommendations of the International Working Group for Diagnosis, Standardization of Response Criteria, Treatment Outcomes, and Reporting Standards for Therapeutic Trials in Acute Myeloid Leukemia. *J Clin Oncol*. Dec 15 2003;21(24):4642-9. doi:10.1200/jco.2003.04.036

[8]. Percival ME, Lai C, Estey E, Hourigan CS. Bone marrow evaluation for diagnosis and monitoring of acute myeloid leukemia. *Blood Rev*. Jul 2017;31(4):185-192. doi:10.1016/j.blre.2017.01.003

[9]. Scrucca L, Santucci A, Aversa F. Competing risk analysis using R: an easy guide for clinicians. *Bone Marrow Transplant*. Aug 2007;40(4):381-7. doi:10.1038/sj.bmt.1705727

[10]. Han G, Schell MJ, Kim J. Improved survival modeling in cancer research using a reduced piecewise exponential approach. *Stat Med*. Jan 15 2014;33(1):59-73. doi:10.1002/sim.5915

[11]. Herold T, Rothenberg-Thurley M, Grunwald VV, et al. Validation and refinement of the revised 2017 European LeukemiaNet genetic risk stratification of acute myeloid leukemia. *Leukemia*. Dec 2020;34(12):3161-3172. doi:10.1038/s41375-020-0806-0
